# Insights into the biosynthesis pathway of phenolic compounds in microalgae

**DOI:** 10.1016/j.csbj.2022.04.019

**Published:** 2022-04-20

**Authors:** Angelo Del Mondo, Clementina Sansone, Christophe Brunet

**Affiliations:** a Stazione zoologica Anton Dohrn, sede Molosiglio Marina Acton, via ammiraglio F. Acton, 55., 80133 Napoli, Italy; b Institute of Biomolecular Chemistry, CNR, via Campi Flegrei 34, Pozzuoli 80078, Na, Italy

**Keywords:** Polyphenols, Flavonoids, Microalgae, Blue biotechnology, *In silico* analysis

## Abstract

Among the most relevant bioactive molecules family, phenolic compounds (PCs) are well known in higher plants, while their knowledge in microalgae is still scarce. Microalgae represent a novel and promising source of human health benefit compounds to be involved, for instance, in nutraceutical composition. This study aims to investigate the PCs biosynthetic pathway in the microalgal realm, exploring its potential variability over the microalgal biodiversity axis. A multistep *in silico* analysis was carried out using a selection of core enzymes from the pathway described in land plants. This study explores their presence in ten groups of prokaryotic and eukaryotic microalgae.. Analyses were carried out taking into account a wide selection of algal protein homologs, functional annotation of conserved domains and motifs, and maximum-likelihood tree construction. Results showed that a conserved core of the pathway for PCs biosynthesis is shared horizontally in all microalgae. Conversely, the ability to synthesize some subclasses of phenolics may be restricted to only some microalgal groups (i.e., Chlorophyta) depending on featured enzymes, such as the flavanone naringenin and other related chalcone isomerase dependent compounds.

## Highlights

• Microalgal PCs are important bioactive molecules beneficial for human health.
• Bioinformatic comparative exploration predicts PCs synthesis in microalgae.
• Ten groups of prokaryotic and eukaryotic microalgae reveal a conserved pathway core.
• Featured PCs can be restricted to diverse microalgae due to ecological implications.

## Graphical abstract

**Figure:**
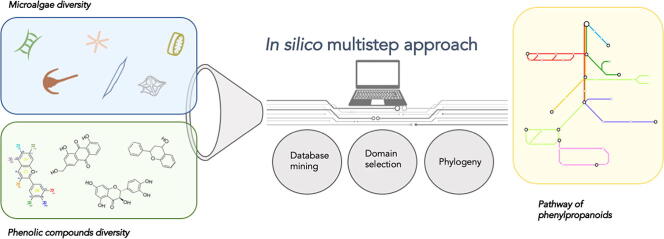

## 1 Introduction

Phenolic compounds (PCs) represent the largest group of secondary metabolites in plants, ranging from simple aromatic rings to more complex molecules and comprising flavonoids, phenolic acids, tannins, lignans or coumarins. They cover a broad range of functions, and are involved in response against environmental stresses, such as heavy metals (e.g., chromium, aluminium), salinity, temperature increase (33–38 °C), pesticides, drought or UV radiations [1], [2], [3], [4], [5], [6]. They also improve nutrient uptake thanks to metallic ions chelation capacity, with the synthesis and exudation of PCs supporting iron acquisition and root-ferric reductase activity [7]. Moreover, it has been observed that plant PCs (e.g., 5-O-caffeoylquinic acid, cinnamic acid, 4-methoxy-cinnamic acid hexoside, K-3-O rutinoside, Q-3-O-rutinoside, Q-3-O-glucoside and Q-3-O-glucuronide) might positively affect mycorrhizal fungi and bacterial root colonizers [8]. PCs do also act as allelochemicals against invading organisms as weeds or pathogens e.g., herbivores, nematodes, phytophagous insects, fungal and bacterial pathogens [9], [10], [11], [12], [13].

**Fig. 1:**
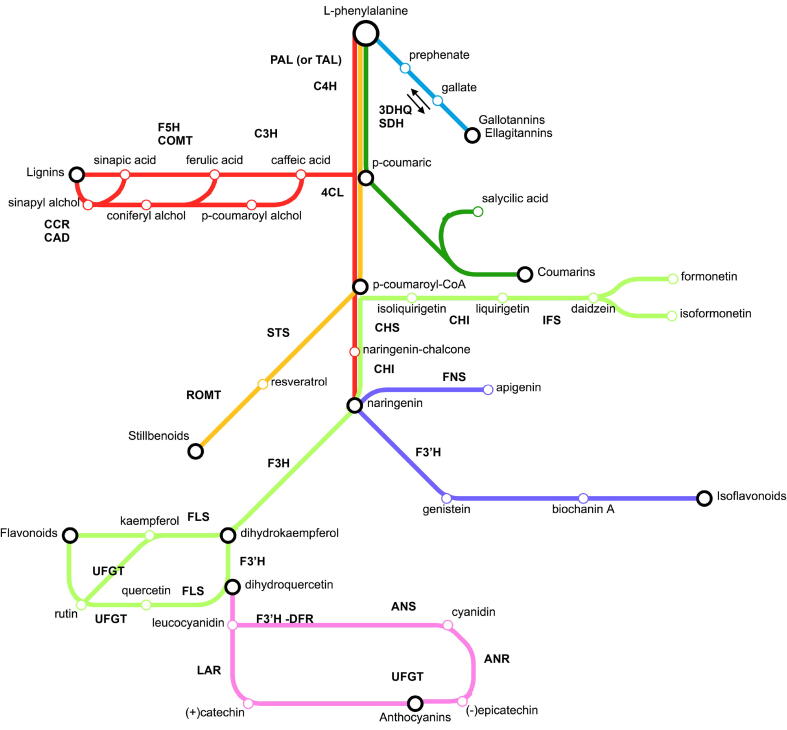
Major routes in phenylpropanoid/flavonoid pathway: red line: lignins; blue line: gallotannins and elagitannins; dark-green line: coumarins; orange line: stillbenoids; purple line: isoflavonoids; light-green line: flavonoids (flavanols, 3-flavonols); pink line: catechins, anthocyanins. (For interpretation of the references to color in this figure legend, the reader is referred to the web version of this article.)

In plants, PCs derive from the biosynthetic pathway of phenylpropanoids, starting with phenylalanine and tyrosine, these two amino acids providing from the shikimic acid and malonic acid pathways [14]. The upstream pathway of phenylpropanoids starts with the transformation of the aminoacid L-phenylalanine by the phenylalanine ammonia lyase (PAL) enzyme, providing a large panel of simple molecules which are then involved in the formation of a plethora of products [15]. The latter includes the hydroxybenzoates (C6-C1) and hydroxycinnamates (C6-C3), involved in the production of the salicylic acid and in the biosynthesis of coumarins ([15], dark-green route in Fig. 1), gallic acid and its derived tannins in the prephenate pathway ([15], blue route in Fig. 1). The main building blocks for lignin biosynthesis are monolignols i.e., hydroxy-cinnamyl alcohols, coniferyl alcohol, and sinapyl alcohol, with typically minor amounts of p-coumaroyl alcohol ([16], green and red routes in the Fig. 1). The downstream flux in the biosynthesis of lignins is essentially regulated by the enzymes CCR1/2 and CAD in plants [17]. Flavonoids synthesis (orange, light-green and purple routes in Fig. 1) starts from naringenin synthetized thanks to the enzymes chalcone synthase and chalcone isomerase (CHS and CHI, [18]). Flavonoids are the largest class of phenylpropanoids in plants, with a basic structure made of two aromatic rings (one from phenylalanine and the other one from the condensation of three malonic acids) linked by three carbons [18]. The main flavonoid classes are the flavones, flavonols, and isoflavones. Flavones (e.g., apigenin, luteolin) and flavonols (e.g., kaempferol, quercetin) are flavonoids absorbing light in the UV region [19], [20]. Anthocyanins are water-soluble pigments belonging to the flavonoids’ family. They are normally stored in the vacuole where they react to the presence of acid residues with variations in colour. In plants, the synthesis of proanthocyanidins branches off the flavonoid pathway from either 2,3-cis or -trans flavanols and is initiated by the dihydroflavonol 4-reductase (DFR), producing leucoanthocyanidin [21]. The key enzyme anthocyanidin synthase (ANS) can later form the intermediate anthocyanidins. Anthocyanidin reductase (ANR) or leucoanthocyanidin reductase (LAR) can operate the conversion into (+)-catechin and (−)-epicatechin, respectively [22], [23]. The latter are then converted into anthocyanins and tannins ([21], pink route in Fig. 1). It is still not clarified whether their polymerization proceeds enzymatically or not [22], [24]. Although studies reported the PCs biosynthetic pathway in higher plants [15], or in other organisms, such as lichens [25], bryophytes [26], ferns [27], [28] or knowledge in algae is almost missing [29], [30], [31]. Algae, from the tiny single cell (microalga) to multicellular seaweeds (macroalga), include prokaryotic cyanobacteria and eukaryotes belonging to diverse evolutionary lineages. Scientific and applied interests in algal biology are growing in the last decade pulled by the “blue biotechnology” development. Indeed, microalgae are attractive being small organisms with fast growth rate, low double time, low nutrient requirement, and able to synthetize secondary metabolites under stress condtions for instance. Microalgae represent a relevant source of bioactive compounds and can become a biofactory for the production of compounds suitable for the market, for instance, related to human health maintenance or protection. In this panorama, PCs are of interest [31], such as in diverse industry sectors such as in nutraceutical product formulation as therapeutic agents for diabetes or cancer, in food as additives and preservatives, in cosmetics as UV-protection and antioxidant agents, and in the textile industry [32]. However, more investigations are required in microalgae being still understudied, conversely to microalgal carotenoids for instance which already fill the industrial market (e.g, astaxanthin, [33]).

This study aims to decipher the biosynthetic pathway of PCs in microalgae advancing an *in silico* analysis on a selection of core enzymes retrieved from the key steps of the biosynthesis of PCs in plants (as presented above, Fig. 1) and exploring the synthetic routes of PCs in microalgae. Yet, this investigation undertakes a comparative analysis between different microalgal divisions. This analysis consisted in i) retrieving putative algal protein homologs of selected core enzymes in the phenylpropanoid pathway, ii) annotating functional domains in each enzyme class, iii) highlighting conserved aminoacidic motifs into the selected domains of interest, and iv) constructing maximum-likelihood phylogenies.

## 2 Materials and methods

### 2.1 Dataset of protein sequences and screening for homologs

Twenty-nine core enzymes of phenylpropanoid/flavonoid pathway were selected from the Kyoto Encyclopedia of Genes and Genomes (KEGG) reference pathway in plants (Table 1).

**Table 1:** Enzyme list. Full description of the 29 core enzymes of phenylpropanoid/flavonoid pathway selected for this study. Plant queries and EC annotation were retrieved from Kyoto Encyclopedia of Genes and Genomes - KEGG database.

Phenylpropanoid/flavonoid core enzymes	Query ID	Plant species	Precursor	Product	EC number	Class
PAL phenylalanine ammonia-lyase	NP_181241; NP_187645; NP_190894; NP_196043.2	*Arabidopsis thaliana*	phenylalalnine	cinnamic acid	EC:4.3.1.24	Lyases
TAL tyrosine ammonia-lyase	XP_015625120	*Oryza sativa*	tyrosine	p-coumaric acid	EC:4.3.1.25	Lyases
C4H *trans*-cinnamate 4-monooxygenase	XP_024156591; NP_180607	*Rosa chinensis; Arabidopsis thaliana*	cynnamoyl-CoA	various	EC:1.14.14.91	Oxidoreductases
COMT caffeic acid 3-O-methyltransferase	XP_024166330; NP_001319057.1	*Rosa chinensis; Arabidopsis thaliana*	caffeic acid	ferulic acid	EC:2.1.1.68	Transferases
C3H coumarate 3-hydroxylase	AAY54293.1	*Ginkgo biloba*	p-coumaric acid	caffeic acid	EC:1.14.14.1	Oxidoreductases
C3′H C3-convertase	O22203.1	*Arabidopsis thaliana*	p-coumaroyl-CoA	caffeoyl-CoA	EC 1.14.14.96	Oxidoreductases
F5H ferulate 5-hydroxylase	AAF78943.1	*Arabidopsis thaliana*	ferulic acid	5-hydroxyferulic acid	EC:1.14.14.B13	Oxidoreductases
4CL 4-coumarate--CoA ligase	NP_175579; NP_176482.1; NP_188760.3; NP_188761.1; NP_192425.1	*Arabidopsis thaliana*	p-coumaric acid	p-cumaroyl-CoA	EC:6.2.1.12	Ligases
CCR1 cynnamoyl coA reductase	AAG46037.1	*Arabidopsis thaliana*	cinnamaldehyde	cinnamoyl-CoA	EC:1.2.1.44	Oxidoreductases
CCR2 cynnamoyl coA reductase	AAG53687.1	*Arabidopsis thaliana*	cinnamaldehyde	cinnamoyl-CoA	EC:1.2.1.44	Oxidoreductases
CAD cinnamyl-alcohol dehydrogenase	NP_177412.1; NP_179765.1; NP_179780.1	*Arabidopsis thaliana*	various	various	EC:1.1.1.195	Oxidoreductases
CHS chalcone synthase	NP_001268064; XP_002264989.1; XP_002272129.2; XP_003634064.1; XP_010655919.1; XP_010662500.1	*Vitis vinifera*	p-cumaroyl-CoA	naringenin-chalcone	EC:2.3.1.74	Transferases
STS stilbene synthase	NP_001268046.1	*Vitis vinifera*	p-coumaroyl-CoA	resveratrol	EC:2.3.1.95	Transferases
ROMT *trans*-resveratrol di-O-methyltransferase	B6VJS4.2	*Vitis vinifera*	resveratrol	pterostilbene	EC:2.1.1.240	Transferases
CHI chalcone isomerase	NP_001268033.1; XP_002280158.1	*Vitis vinifera*	naringenin-chalcone	naringenin	EC:5.5.1.6	Isomerases
FNS flavone synthase I	Q7XZQ8	*Vitis vinifera*	various	apigenin, luteolin	EC:1.14.20.5	Oxidoreductases
FNS flavone synthase II	XP_024155936; XP_024155939.1; XP_024155941.1	*Rosa chinensis*	various	various	EC:1.14.19.76	Oxidoreductases
IFS isoflavone synthase	Q9SXS3.1	*Glycyrrhiza echinata*	various	various	EC:1.14.14.87	Oxidoreductases
F3H flavanone3-hydroxylase	NP_190692	*Arabidopsis thaliana*	various	various	EC:1.14.11.9	Oxidoreductases
DFR dihydroflavonol 4-reductase	P51102.2	*Arabidopsis thaliana*	various	various	EC:1.1.1.219	Oxidoreductases
F2H 2′-hydroxyisoflavone reductase	P52575.1	*Medicago sativa*	various	various	EC:1.3.1.45	Oxidoreductases
UFGT (UDP flavonoid glycosyltransferase)	P16165.1	*Zea mays*	cyanidin, delphinidin, etc	various	EC:2.4.1.115	Transferases
FS flavonol synthase	NP_001190266.1; XP_015624815.1	*Arabidopsis thaliana; Oryza sativa*	dihydrokaempferol	kaempferol	EC:1.14.20.6	Oxidoreductases
F3′H flavanone3′-hydroxylase	NP_196416	*Arabidopsis thaliana*	kaempferol	quercetin	EC:1.14.14.82	Oxidoreductases
LAR leucoanthocyanidin reductase	XP_015630916	*Oryza sativa*	leucocyanidin	(+)-catechin	EC:1.17.1.3	Oxidoreductases
ANS anthocyanidin synthase	NP_001031700.1	*Arabidopsis thaliana*	various	various	EC:1.14.20.4	Oxidoreductases
ANR anthocyanidin reductase	XP_015635209.1; XP_015637098; XP_015637099.1	*Oryza sativa*	cyanidin	(−)-epicatechin	EC:1.3.1.77	Oxidoreductases
3-dehydroquinate dehydratase / shikimate dehydrogenase	XP_002884573	*Arabidopsis lyrata*	3-dihydroshikimate	shikimate	EC:4.2.1.10	Lyases
polyphenol oxidase	NP_001268045.1; XP_010647098.2	*Vitis vinifera*	various	various	EC:1.10.3.1	Oxidoreductases

A BLASTp search (v. 2.7.1.) [34] using the enzymatic classes as queries against the entire UniProt Swiss-Prot protein database [35] was performed to detect orthologs of phenylpropanoid and flavonoid pathway enzymes within different algal divisions. Analytical outputs regarded the manually annotated and reviewed protein belonging to Cyanobacteria, Bacillariophyta, Chlorophyta, Cryptophyta, Rhodophyta, Dinophyta, Euglenozoa, Haptophyta, Eustigmatophyta and Phaeophyta-Xanthophyta clades. A BLASTp search against the UniProt TrEMBL protein database [36] was also done, including the computationally analyzed records – not yet annotated - belonging to the same clades. The best hit matches for each species were retained for down-stream analyses. Using a subset of representative taxa in each microalgal division (Table 1), a heatmap from the matrix of normalized BLAST scores was built using Graphpad Prism 8.0 software. A principal component analysis (PCA, using a variance–covariance matrix) was carried out on the same matrix using PAST 4.04 software [37].

### 2.2 Multiple alignments and Maximum-Likelihood (ML) phylogenetic analysis

Sequences retrieved by BLASTp search in the UniProt TrEMBL protein database were first aligned with MAFFT software v7.397 [38] applying default parameters to generate phylogenetic trees for the core enzymes. Alignments were then cleaned with TrimAl v1.4 [39] using the values 0.25 for gap threshold, 0.25 for residue overlap threshold, and 0.9 for sequence overlap. The best-fit model of molecular evolution for each dataset was selected with ModelFinder, implemented in IQ-TREE v1.6.11 [40] and operating the corrected Akaike Information Criterion (AICc). Maximum-likelihood trees were constructed with FastTree v2.1.11 [41] applying default parameters. All trees were visualized and edited using iTOL v.5 [42].

### 2.3 Functional annotation and selection of Domains of Interest (DOIs)

Functional annotation was performed using the software InterProScan (version 5.33) [43] to retrieve the InterPro Domains associated to the queries together with their coordinates along the investigated algal sequences collected in the UniProt database. A selection of the domains of interest (DOIs) was manually curated, discarding non-characteristic or ambiguous domains, onlyretaining the functional ones. For each best hit protein satisfying quality criteria and DOIs selection, only the sequence region corresponding to the coordinates of the detected InterPro Domain was retained. Followingly, the retained domain sequences detected for each enzymatic class were used to identify the enrichment motifs among classes by MEME and STREME tools of MEME suite software (v. 5.3.3.) [44]. Higher plants sequences collected by the previous BLAST search and subjected to same data treatment were then employed as reference sequences for STREME consensus analysis. Relevant MEME motifs in selected DOIs were added as visual data to ML-phylogeny, whereas positive algal DOIs in STREME analysis were then parsed on p-value score and percent of positive algal sequences (Table A.1).

## 3 Results and discussion

**Fig. 2:**
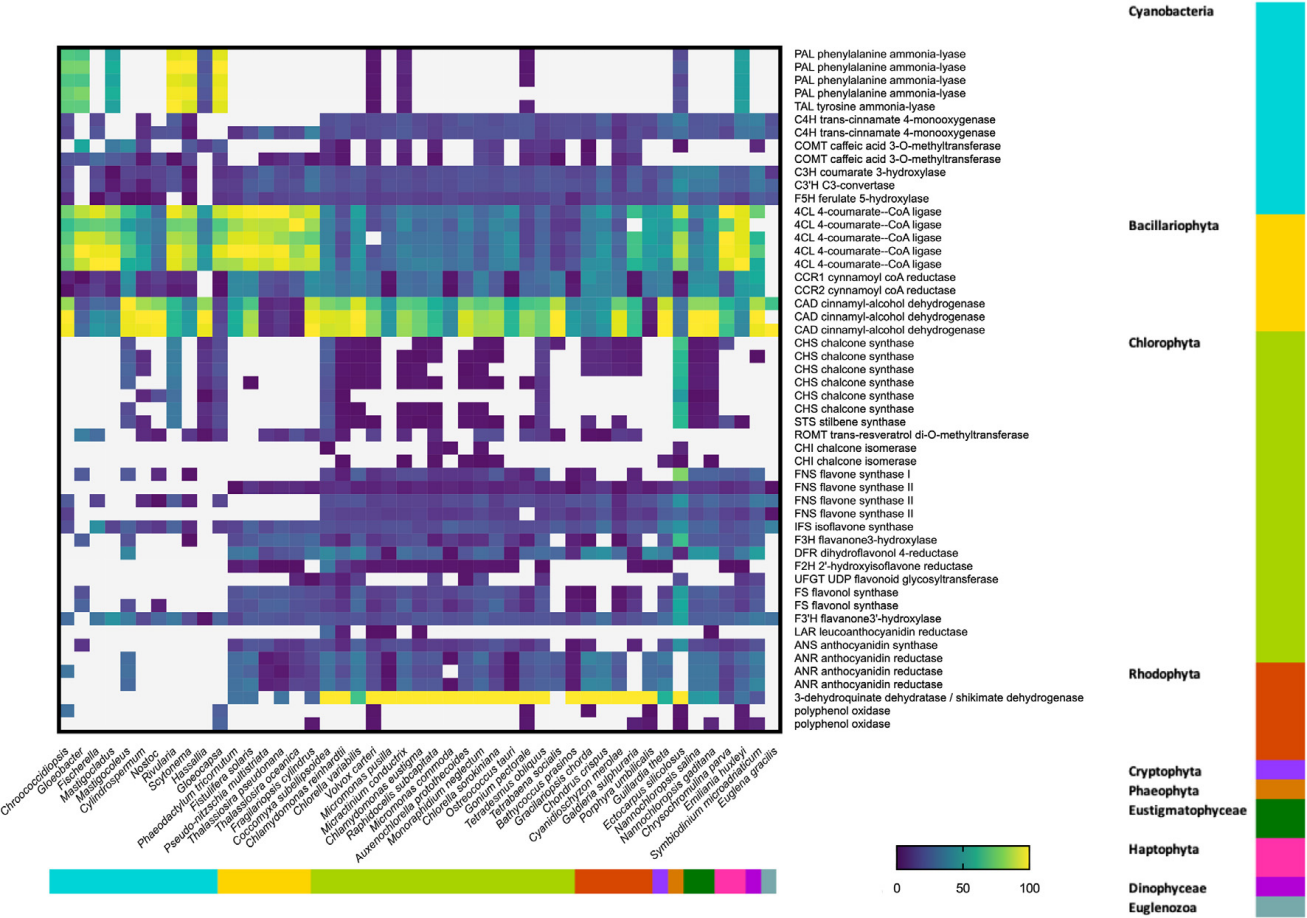
Heatmap build by normalized BLASTp scores obtained from plant entries of 29 selected core enzyme in phenylpropanoid pathway using. A subset of 47 algal taxa.

**Fig. 3:**
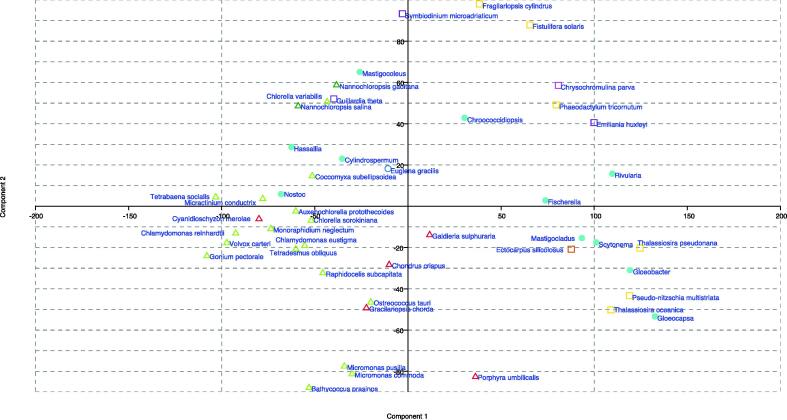
PCA of normalized BLASTp scores obtained from plant entries of 29 selected core enzymes in phenylpropanoid pathway. Cyanobacteria (dots), primary endosymbiotic event (triangles) and secondary endosymbiotic event (squares) eukaryotic algae are plotted following the colour legend in Fig. 2. Tertiary endosymbiotic event alga *Euglena* is marked by a void circle.

The BLAST scores contribution of the twenty-nine core enzymes (Table 1) among the different microalgal groups showed a great variability (Fig. 2), confirmed by the PCA (axis 1: 46.4%; axis 2: 18.2% of the variability, Fig. 3). Cyanobacteria was heterogeneous and dispersed radiantly among quadrants (Fig. 3**)**. While ancestral prokaryotes, Cyanobacteria display the greatest variability in the pathway, revealing their evolution with time [45], [46]. Primary (Chlorophyta, Rhodophyta, Eustigmatophyta) and secondary (Bacillariophyta, Cryptophyta, Charophyta, Haptophyta, Dinophyta) endosymbiotic algae displaced toward opposite direction, suggesting that the endosymbiotic gene transfer (EGT) played a fundamental role as driver in the evolution of phenylpropanoids in different phyla. The only tertiary endosymbiotic alga in the dataset, *Euglena*, encountered massive gene loss, strongly decreasing its weight in the analysis (Fig. 3). Following this first descriptive overall approach, the analysis of key enzymes building algae-based maximum likelihood phylogenetic trees was then performed to compare the biosynthetic pathway of the main PCs amongst microalgae.

### 3.1 The routes for lignins, coumarins and gallic acid derived-tannins

#### 3.1.1 Phenylalanine ammonia lyase (PAL)

In plants, the enzymes PAL (phenylalanine ammonia lyase) or TAL (tyrosine ammonia lyase) catalyse the non-oxidative deamination of phenylalanine (or tyrosine) to *trans*-cinnamate and direct the carbon flow from the shikimate pathway to the various branches of the general phenylpropanoid metabolism (Fig. 1). PAL activity produces *trans*-cinnamic acid, whose derivatives have been detected in several algal divisions [31]. While the activity of PAL enzyme has been demonstrated *in vitro* in cyanobacteria [47], to the best of our knowledge no experimental demonstration has been provided in eukaryotic algae. Structural data of PALs are available for the cyanobacteria *Anabaena* and *Nostoc* [48] and for the streptophyte algae *Klebsormidium* and *Nitella* [30]. In Cyanobacteria, PALs are smaller in size than their eukaryotic counterparts; however, they show similar substrate selectivity and kinetic activity toward L-phenylalanine over L-tyrosine [48]. Structure elucidation by protein X-ray crystallography confirmed that the two cyanobacterial PALs are similar in tertiary and quaternary structure to plant and yeast PALs [48].

**Fig. 4:**
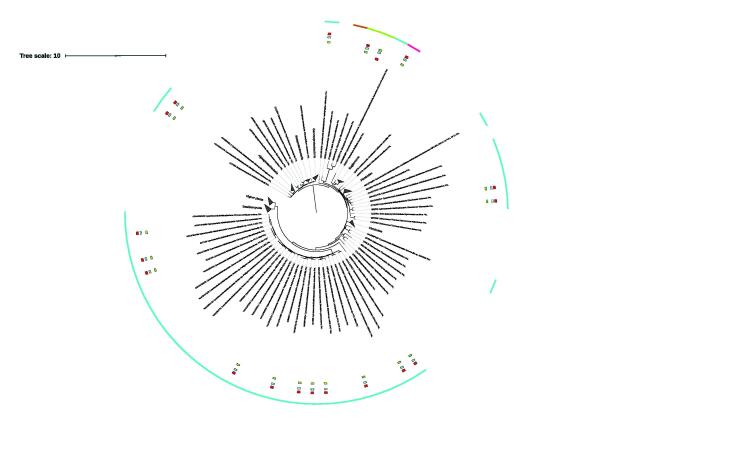
ML-tree for PAL enzyme. Phylogeny was obtained starting from 236 protein sequences. Thicker clades indicate node support >75%. Green, cyan and magenta boxes represent motifs retrieved by MEME analysis of algal sequences within the domain IPR001106. Algal divisions are indicated as color arches, following the legend presented in Fig. 2. (For interpretation of the references to color in this figure legend, the reader is referred to the web version of this article.)

In our study, the occurrence of PAL (or TAL) homologs in algae showed disparate hits: its presence was recorded in the brown seaweed *Ectocarpus* and in the haptophyte *Emiliania huxleyi,* and to a lesser extent within Cyanobacteria and Chlorophyta, as shown in Fig. 2 and Fig. 4. This unexpected feature addresses the question on the initiation of the biosynthetic phenylpropanoid pathway in the remaining considered algal divisions, e.g. in Bacillariophyta or Rhodophyta, in which phenolic compounds production is however well documented. With the exception of *Ectocarpus,* PAL deficiency appears to be common to all secondary endosymbiotic algae, while it could have been partly loss in Cyanobacteria and Chlorophyta.

PAL sequences from *Arabidopsis thaliana* and a TAL sequence from *Oryza sativa* (Table 1) were employed to build a ML phylogeny tree using both enzymes which own an aromatic amino acid lyase domain (IPR001106) (Table A.1). The Maximum-likelihood (ML) phylogenetic analysis of PAL in the diverse microalgal groups (Fig. 4) agrees with the previously reported phylogenetic observations [49], [50]. The brown alga *Ectocarpus* and the two Chlorophytes *Micractinium* and *Gonium* shared a very robust node (100) and cluster with the archaeal *Halobacterium salinarum* (77.5). Notably, conserved motifs of PAL-domain in *Gonium* were reversed, lone case among the MEME analyzed sequences, while *Ectocarpus* did not show reliable motif conservation. The haptophyte *Emiliania* appeared to be closely related to the Euglenozoa clade (98.5) and both shared a robust node with *Candidatus Marinamargulis bacterium* (98.3). For this Carbon-Nitrogen lyase, cyanobacterial species belonged to diverse clusters composed by various classes of Eubacteria, probably accounting for duplication and loss among prokaryotic algae and hinting for a hidden paralogy.

#### 3.1.2 Shikimate dehydrogenase (SDH) and 3-dehydroquinate dehydratase (3-DHQ)

Plant SDH enzymes are fused to dehydroquinate dehydratases to form a bifunctional SDH/DHQ. This enzyme catalyzes the dehydration of 3-dehydroquinate to 3-dehydroshikimate (3-DHS) and the reversible reduction of the latter into shikimate [51]. The 3-dehydroquinate can be converted to quinic acid, an important precursor for aromatic amino-acids and for other molecules, some with notable herbicidal or pharmacological activities [52]. It is also noteworthy that dehydroshikimate is the precursor for abscisic acid (ABA) hormone and protocatechuic acid, both reported in microalgae [31], [53]. Protocatechuic acid has been identified in different microalgal groups, i.e., the diatom *Phaeodactylum tricornutum*, the Eustigmatophyte *Nannochloropsis gaditana* and the green alga *Nannochloris* sp. and *Tetraselmis suecica* [54]. Interestingly, protocatechuic acid was reported as growth promoter for the microalga *Euglena gracilis* [55]. Use of protocatechuic acid might open new cost-effective strategy for improving the microalgal productivity in large-scale cultivations. The plant bifunctional SDH/DHQ enzyme presented the highest similarity score within Chlorophyta and Rhodophyta groups (Fig. 2). A weaker although substantial similarity score was reported for diatoms, *Chrysochromulina*, *Emiliania*, and *Symbiodinium* (Fig. 2). No SDH/DHQ enzyme hit was retrieved in Cyanobacteria and *Euglena.* Interestingly, microalgae which do not possess PAL enzyme hold 3-DHQ enzyme with the higher scores (Fig. 2), opening a question on the putative role of SDH/DHQ enzymes in the onset of phenylpropanoid pathway. The 3-DHQ algal homologs contain a domain associated with prokaryotic shikimate-5-dehydrogenase, a Carbon-Oxigen lyase in the SDH superfamily (IPR011342) (Table A.1). This domain, involved in NADP-dependent reduction of 3-dehydroshikimate to shikimate is present in several algal phyla, even though patchy distributed among three clades (Fig. A.1): a) *Ectocarpus*, Eustigmatophyta, Bacillariophyta; b) Cyanobacteria; c) Chlorophyta, Rhodophyta, Cryptophyta, Haptophyta, and a candidate cyanobacterial division.

#### 3.1.3 4-Coumarate ligase (4CL)

The co-A ligase 4CL and the hydroxylase C4H are upstream enzymes for the synthesis of flavonoids, stillbenoids, coumarins, lignins, catechins, and aurones through the intermediate p-coumaroyl-CoA.

In plants, 4-coumarate ligase 4CL catalyzes the stepwise conversion of hydroxycinnamic acids into the corresponding CoA esters via the intermediate AMP derivatives. A sequence similarity between prokaryotic and eukaryotic 4CL has been reported [56] and both does function via an ATP-dependent covalent binding of AMP to their substrate.

**Fig. 5:**
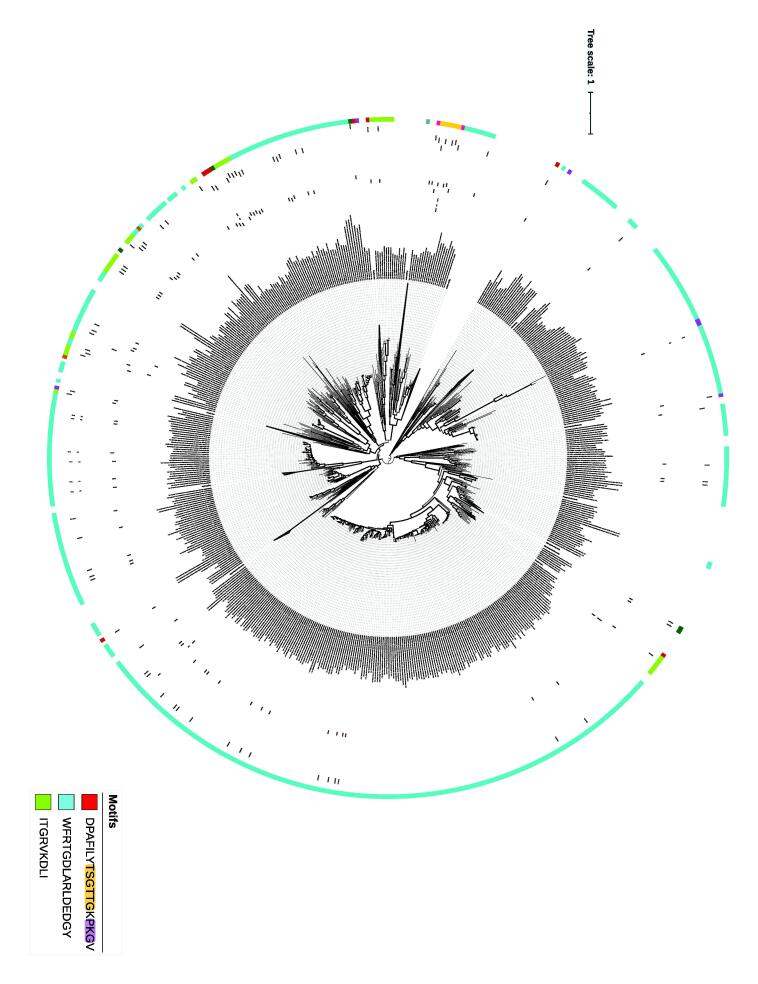
ML-tree for 4CL enzyme. Phylogeny was obtained starting from 882 protein sequences. Thicker clades indicate node support >75%. Green, cyan and magenta boxes represent motifs retrieved by MEME analysis of algal sequences within the domain IPR000873. The Ser/Thr/Gly-rich trait and a conserved Pro-Lys-Gly triplet are highlighted in the red box. Algal divisions are indicated as color arches, following the legend presented in Fig. 2. (For interpretation of the references to color in this figure legend, the reader is referred to the web version of this article.)

The precursor p-coumaroyl-CoA is necessary for the synthesis of several flavonoid derivatives (i.e., 5-deoxyflavonoids or dihydroflavonols) present in the major algal divisions of cyanobacteria, diatoms and green algae [31]. The BLAST search for 4CL revealed substantial similarity score in cyanobacteria, diatoms, and haptophytes (Fig. 2). Although the haptophytes *Emiliania* and *Chrysochromulina* as well as diatoms underwent a secondary endosymbiosis event, only diatoms showed a true protein orthology for 4CL in our study (Fig. 5). Haptophytes and diatoms showed the highest similarity scores for 4CL with respect to higher plants (Fig. 2**)**. The common node between these two groups and green algae in the 4CL-based phylogenetic tree might reveal a green algal ancestor in the evolution of plant 4CL enzyme (Fig. 5). Remarkably, the characteristic domain IPR000873 (owned by all sequences in our phylogeny) highlighted a Ser/Thr/Gly-rich trait followed by a conserved Pro-Lys-Gly triplet (see red box in Fig. 5) that remains highly and stably conserved within all the analysed algal taxa.

#### 3.1.4 Cinnamoyl Co-A reductase (CCR) and cinnamoyl alcohol dehydrogenase (CAD)

The enzymes CCR1/2 and CAD involved in lignin biosynthesis in plants [17] appear conserved in algae, with the only exception of the tertiary endosymbiotic alga *Euglena* (Fig. 2).

Plant CCR 1 and 2 convert hydroxycinnamoyl CoA esters to their corresponding cinnamyl aldehydes in monolignol biosynthesis and differ by substrate affinity towards feruloyl-CoA and caffeoyl-CoA, respectively [57]. Algal homologs for CCR displayed a cyanobacterial ancestry (Fig. A.2) and subsequent gene duplication would be supported by paralogy of Bacillariophytes, Haptophytes and Chlorophytes.CADs are zinc-dependent dehydrogenases and catalyze the last step of monolignol biosynthesis by reducing cinnamaldehyde into cinnamyl alcohols prior to their transport through the membranes and polymerization in the cell wall [58]. In higher plants three classes of CADs are present, following a scheme of duplication and extinction with complex spatial and temporal regulation [59]. Lignin, lignin-like compounds, and sporopollenin were found in the cell wall of seaweeds, such as in *Coleochaete* (Chlorophyta) [60], *Cystoseira* (Phaeophyta) [61] and *Sargassum* (Phaeophyta) [62] as well as in microalgae [63], [64], [65]. It has been recently elucidated that sporopollenin precursors originate in the phenylpropanoid pathway in plants, following a scheme that is superimposed to canonical lignin biosynthesis [66]. The discovery of lignified cell wall in the seaweed *Calliarthron* enlightened the possibility of convergent evolutionary history of this trait, given the early separation among green and red algal lineages [67]. Recently, a hypothesis of lateral gene transfer from bacteria to algae has been proposed to explain the presence of lignin-related compounds in seaweeds [68].

**Fig. 6:**
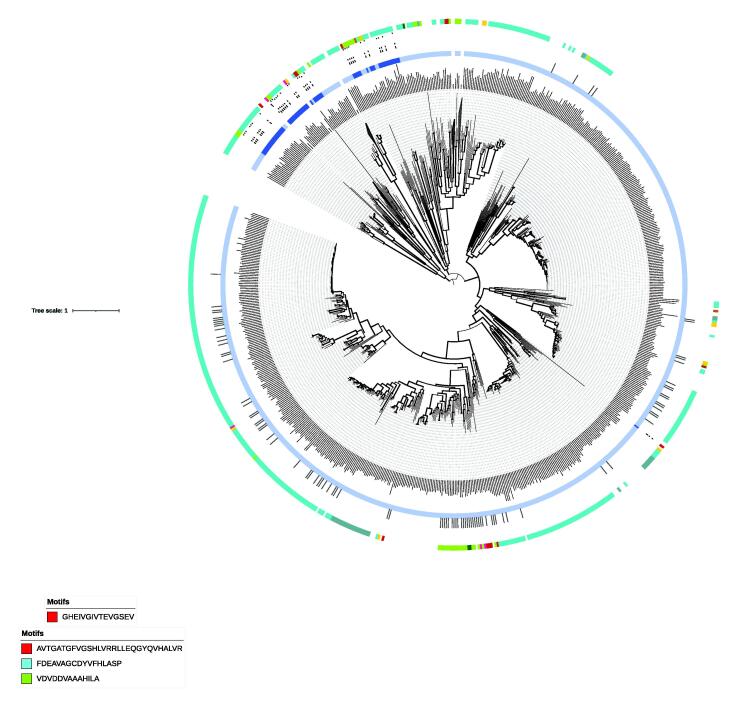
ML-tree for CAD-DFR enzymes. Phylogeny was obtained starting from 722 CAD and 109 DFR protein sequences. Thicker clades indicate node support >75%. At the end of leaves, dark and light blue colors differentiate DFR and CAD sequences, respectively. Red bars and green, cyan and magenta boxes represent motifs retrieved by MEME analysis of algal sequences within the respective domains IPR002328 and IPR001509. Algal divisions are indicated as color arches, following the legend presented in Fig. 2. (For interpretation of the references to color in this figure legend, the reader is referred to the web version of this article.)

In our study, beside red and green algae, also diatoms (possessing a silica cell wall), cyanobacteria and *Euglena* showed remarkable similarity scores for CAD (Fig. 2). Very few studies investigated the presence of lignins in microalgae [69]. Lignin has been reported in *Staurastrum* sp. and in a mixture of cyanobacteria and eukaryotic microalgae [69]. This surprising feature might indicate that in microalgae, lignin precursors and CAD activity may play another role that the deposition of lignin in the cell wall. Hydrolized lignin and lignin-derived products from plants are known for their capacity to inhibit algal growth [70], [71]. It is noteworthy that lignin might have important biotechnological potential e.g., for biopolymers, heavy metal removal or energy [72], requiring more investigations in microalgae. The phylogeny analysis revealed that a large group of OTUs including also plant and bacteria shares the domain IPR002328 (Zinc-containing 'long-chain' alcohol dehydrogenases) (red star clade in Fig. 6). Within the red star clade, a great group of eubacteria clusters together with cyanobacteria and eukaryotic algae, suggesting a common prokaryotic origin for this enzyme.

Dihydroflavonol 4-reductase– DFR is an oxidoreductase involved in the diversification of flavonoids starting from dihydrokaempferol or dihydroquercetin, both flavonols reported in algae [31]. Both CAD and DFR (Table 1) are oxidoreductases acting on CH-OH group of donors with NAD+ or NADP+ as acceptor; although they catalyze different reactions, the two enzymes own a very similar asset. Previous reports indicated significant CAD homologies with dihydroflavonol-4-reductases from various plant species [73]. It is known that minor changes in DFR sequence may determine alteration in substrate specificity [74]. DFR sequences included in our phylogeny do also present the NADP-binding domain (IPR001509) required for the enzymatic activity (Table A.1). The CAD sequences interspersed in the DFR clades were not featured by neither Zinc-containing 'long-chain' alcohol dehydrogenases domain, nor by a NADP-binding domain. Both CAD and DFR enzymes displayed a horizontal distribution among algal divisions (Fig. 2), and the tree topology (Fig. 6**)** hinted that ancestral algal CADs may have diversified into DFRs, establishing separate lignin and flavonoid routes.

### 3.2 Routes for stillbenoids, flavonoids and isoflavones

#### 3.2.1 Chalcone synthase (CHS) and Stillbene synthase (STS)

Chalcone synthase and Stillbene synthase are two transferases involved in the onset of the flavonoid pathway and stillbenoids biosynthesis (i.e., resveratrol), respectively. Stillbenoids display a heterogeneous distribution in the plant Kingdom while not yet revealed in algae [75].

**Fig. 7:**
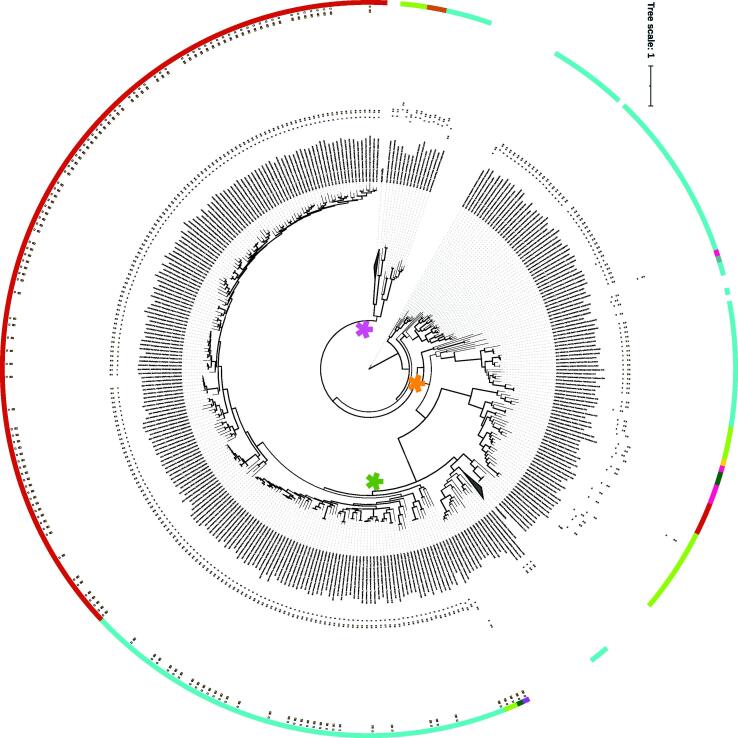
ML-tree for CHS enzyme. Phylogeny was obtained starting from 506 protein sequences. Thicker clades indicate node support >75%. Green, cyan and magenta boxes represent motifs retrieved by MEME analysis of algal sequences within the domains IPR011141 (inner) and IPR004655 (outer). Algal divisions are indicated as color arches, following the legend presented in Fig. 2. (For interpretation of the references to color in this figure legend, the reader is referred to the web version of this article.)

CHS catalysis serves as initial step for flavonoid biosynthesis, associated with the production of chalcones [76]. This enzyme, ubiquitous in higher plants, is known as type III PKS (Polyketide synthase type) in the family of polyketide synthase enzymes (PKS). CHS followed a patchy distribution among algal divisions, displaying the highest similarity scores in the brown macro-alga *Ectocarpus,* although lacking in other groups e.g., diatoms (Fig. 2). The presence of several flavonoid compounds reported in diatoms [77], [29] might indicate that flavanol reduction could be deputed to other oxidoreductase(s) with larger substrate specificity. Yet, it was hypothesized that styrylchromone toxins isolated from the marine cryptophyte *Chrysophaeum taylori* are formed by a type III PKS enzyme [78], while our study did not identify CHS homologs in another marine cryptophyte, *Guillardia theta* (Fig. 2 and Fig. 7).

In the algal CHS-based ML-phylogeny tree (Fig. 7), generally cyanobacteria ranked at a basal position, although a subsequent clusterization did exist in higher clades. This happened as a sister clade in the red algae division (orange star in Fig. 7) as well as in the apical branch containing higher plants as a sister clade of brown algae (pink star in Fig. 7). This distribution would suggest the rise of CHS enzyme more than once in algal divisions, converging with the differences among the widespread flavonoids present in green algae and the more complex flavonoids of red and brown seaweeds [21].

Consensus retrieval for the identified domains indicated the presence of Polyketide synthase type III domain (IPR011141), and a 3-oxoacyl-[acyl-carrier-protein]-synthase3 domain (IPR004655) (Table A.1). The first, deputed to perform three sequential condensation steps with acetate units from malonyl-CoA to form a tetraketide intermediate starting from a CoA-ester, showed horizontal conservation among all microalgal divisions. The second (responsible of the elongation in plant type II fatty acid synthase systems) was only present in a restricted group of algae sharing one common node and including Cyanobacteria, Rhodophyta, Chlorophyta, *Nannochloropsis*, and *Symbiodinium* (green star in Fig. 7).

#### 3.2.2 Chalcone isomerase – CHI

Chalcone isomerase – CHI drives the fundamental step to generate flavanones. Isomerasic activity is relevant for the diversification of flavonoids with e.g., the naringenin-chalcone conversion into naringenin. This colourless flavanone has been reported in the haptophyte *Diacronema lutheri* and in the chlorophyte *Haematococcus pluvialis* [29]. Its distribution appeared heterogeneous among algal divisions (Fig. 2), being absent in cyanobacteria, red algae, and diatoms. The function of CHI, and thereby the ability to synthetize flavanones, could be originated from the green algal lineage, and followingly encountered gene duplication and selective loss, persisting in Chlorophyta, Phaeophyta, Cryptophyta and in the class of Eustigmatophyceae, as well as in higher plants (Fig. A.3). In STREME analysis of retrieved sequences, the DOI of chalcone isomerase domain (IPR016087) showed 100% positive algal sequences in a motif consensus comparison with higher plants (Table A.1).

Flavanones can be further converted to flavones or to dihydroflavonols thanks to the flavone synthase II (FNS) or to the flavanone3-hydroxylase (F3H) enzyme, respectively [18].

These products can be then converted to flavonols (through flavonol synthase (FLS) enzyme activity), or to leucocyanidin (through flavanone3′-hydroxylase (F3′H) or dihydroflavonol 4-reductase (DFR) enzyme activities), entering into the anthocyanidin pathway [18]. A large subset of enzymes deputed to the modification and interconversion of flavonoids appear to be evenly present among microalgal divisions, with the only exception of cyanobacteria. However, several flavonoids were found in some cyanobacterial species (e.g., quercetin, rutin, apigenin, daidzein, catechin or epicatechin [31]) addressing the hypothesis of the non-enzymatic production of these molecules or on the presence of other enzymes in cyanobacteria. Isoflavones, such as daidzein or genistein were retrieved in the green microalga *Haematococcus pluvialis,* together with flavones or flavonols [29]. Also, they have been found in the diatom *Phaeodactylum tricornutum* and in the red microaga *Porphyridium* [29]. The lack of information on the distribution in other species of these two isoflavones prevent any taxonomical-derived hypothesis.

### 3.3 Route for anthocyanins

#### 3.3.1 Anthocyanidin reductase (ANR) and leucoanthocyanidin reductase (LAR)

ANR uses cyanidin as a precursor to form (−)-epicatechin, while LAR synthetizes (+)-catechin by transforming leucocyanidin. Both catechin and epicatechin are found in red and brown algae [79], [80], in the diatom *Phaeodactylum tricornutum* [77], in *Euglena gracilis* [81], and in some green algae [80], [81] and cyanobacteria [80], [81], [82]. Catechin and epicatechin present biological interests, being reported as antioxidant and anti-inflammatory [83].

The BLASTp search for DFR, ANS (anthocyanidin synthase) and ANR (anthocyanidin reductase), enzymes necessary for the synthesis of catechin and epicatechin (Fig. 2), is in agreement with previous reported observations in algae; difference regards *Euglena*, in which no substantial hits were retrieved despite experimental reports for both (−)-epicatechin and (+)-catechin [81].

Recent studies hypothesized an algal origin for anthocyanins in plants although its presence in algae remains uncertain [84]. Cyanidin presence in algae has never been revealed, although free and glycosylated catechins were detected in several algal divisions [31]. The analysis of conserved DOIs in ANR and LAR putative algal homologs, reveals the presence of a NAD-dependent epimerase/dehydratase domain (IPR001509) for ANR, and the presence of a NmrA-like domain (IPR008030) for LAR [85] (Table A.1).

#### 3.3.2 UPD-glucose:flavonol 3-O-D glucosyltransferase (UFGT)

Another transferase which proceeds downstream the flavonoid pathway (light-green and pink routes in Fig. 1) is the UPD-glucose:flavonol 3-O-D glucosyltransferase (UFGT). In plants this glucosyltransferase catalyzes the transfer of glucose from UDP-glucose to a flavanol, a step that is necessary to proceed toward anthocyanin pigment biosynthesis. A study reports that the UGT family expanded during the transition from algae (e.g., the chlorophyte *Chlamydomonas reinhardtii*) to vascular plants [86]. UFGT operates the conversion of intermediate anthocyanidins into a variety of anthocyanins, which to date, have not been reported in algae [87]. Only one study reported the presence of cyanidin and malvidin derivatives in the green seaweed *Caulerpa racemosa* [88]. Recent studies point out an ancestral origin of anthocyanins and tannins in algae [89]. However, uncommon purple pigments with a chemical structure closely related to anthocyanins have been rarely found in algae, for instance in the cyanobacterium *Tolypothrix tenuis* [90], or in the Charophyta *Zygogonium ericetorum* [91], [92]. Also, the blue pigment marennine to date retrieved only from the diatom *Haslea ostrearia* might belong to this group [93], [94]. This pigment presents many commercial or biological interests, due to its antioxidant, antibacterial or antiviral activities [94]. The BLAST search for UFGT homologs in algae revealed low identity scores, with substantial hit in *Ectocarpus*, *Chrysochromulina* and partly in diatoms and green algae (Fig. 2). No hits were retrieved in Rhodophyta, Cryptophyta, Eustigmatophyceae and Dinophyceae.

ML phylogeny for *bona fide* algal UFGT (Fig. A.4) did not show significant relation with prokaryotic or eukaryotic non-photosynthetic OTUs. A first subgroup of unicellular green algae clustered intimately with higher plants, while another subgroup revealed more ancestral traits, being distributednear to other microalgae, including cyanobacteria. To date, there is no report of UFGT enzyme activity in algae, even though its functional domain (IPR002213) displays a conserved region among the considered algal sequences (Table A.1).

### 3.4 Branching off the pathway

The chemodiversity of PCs in microalgae is further enhanced by possible further diversifications, such as additional methylation or multiple hydroxylation. These modifications can change the role or the bioactive capacity of PCs without dramatical change on the carbon skeleton.

#### 3.4.1 O-methyltransferases (OMT)

The enzymatic O-methyltransferasic activity is crucial to direct the biosynthesis of lignins with the caffeic acid 3-O-methyltransferase (COMT) or the diversification of stillbenoids, with *trans*-resveratrol di-O-methyltransferase (ROMT). Since stillbenoids are not retrieved in algae, the putative OMT activity of the retrieved homologs relates with the interconversion of phenolic acids starting from caffeic acid (or caffeoyl-CoA) intermediate to generate ferulic acid (or feruloyl-CoA) before further modification by CCR and CAD to synthetize lignin and lignin like compounds and diversify phenolic acids (red route in Fig. 1). Putative COMT homologs were found in a subgroup of green algae positioned as a basal clade in our phylogeny (Fig. A.5). Land plant sequences were located as a sister clade of cyanobacteria and diatoms, both sharing a common node with a wide group of eukaryotic and prokaryotic microalgae. The only two bacterial putative COMT present in the phylogeny, belonging to the genus of photosynthesizing nonsulfur purple bacteria *Rhodobacter*, intimately cluster within the basal chlorophyte clade with a significant node support. This relation could reveal an Horizontal Gene Transferevent for the evolution of OMT in the microalgal world.

#### 3.4.2 Oxidoreductases

Oxidoreductases can extensively increase the degree of diversification of phenolic compounds. The selected oxidoreductases can be grouped in four macro-classes on the basis of EC number, namely: a) *EC 1.1* – Acting on the CH-OH group of donors (i.e., CAD, DFR); b) *EC 1.2* – Acting on the aldehyde or oxo group of donors (i.e., CCRs); c) *EC 1.3* – acting on the CH-CH group of donors (ANR, F2H); 4) *EC 1.14* – paired donors with incorporation of molecular oxygen (i.e., C4H, FS, ANS).

The role of C4H, deputed to the conversion of cinnamic acid into p-coumaric acid (or cinnamoyl-CoA into p-coumaroyl-CoA), may be bypassed in case Tyr is initiator of the pathway, due to the preliminary and necessary conversion of Phe into cinnamic acid [15]. The oxidoreductases C3H, C3′H and F5H further increase the diversity of phenolic compounds acting on a number of substrates [15]. In the Fig. 1, the enzymes C3H, C4H, F5H (red route), IFS, F2H, F3H, FLS (light-green route), FNS (purple route), and ANR, ANS (pink route) are represented.

The algal homologs of the oxidoreductases listed in Table 1 involved in the branching off of the pathway were included in a phylogeny to evaluate possible interrelation (Fig. A.6). The considered oxydoreductases displayed a high degree of inter-dispersion among the different clades, in agreement with results reported for C4H or F5H which show ambiguous orthologs in land plant phylogenies [50].

### 3.5 Extracellular polyphenol oxidases and secretion of phenolic compounds

It is reported that a number of cyanobacteria and green algae are able to degrade phenolic compounds outside the cell wall [95], [96], [97], [98], [99], [100]. In Chlorophyta cultivation, the release of polyphenol oxidases with laccase-like activity in the medium reaches its maximum during the stationary growth phase and seems to increase with copper sulphate administration [101]. This might be of interest for biotechnological remediation of polluted waters [101].

In our study, extracellular polyphenol-oxidase enzymes resulted in spotted hits in several groups of microalgae, including cyanobacteria and green algae, as well as the microalgae *Fragilariopsis* (Bacillariophyceae), *Galdieria* (Rhodophyta), *Nannochloropsis* (Eustigmatophycea), *Emiliania* (Haptophyta), *Symbiodinium* (Dynophyta), and the seaweeds *Porphyra* (Rhodophyta) and *Ectocarpus* (Phaeophyta) (Fig. 2). These findings suggest that the ability to secrete enzymes able to degrade phenolic compounds in the external environment may be present in diverse algal divisions and have profound ecological implications in determining the efficacy of extracellular signalling [102].

For example, it is known that aquatic plants may exert an algicidal allelopathy especially towards cyanobacteria and green microalgae through the secretion of various phenolic compounds [103].

This feature depends on the considered species and/or compounds. For instance, Wang and co-workers [104] observed that submerged macrophytes inhibited the growth of the microalgae *Microcystis aeruginosa* (Cyanobacteria) and *Pseudokirchneriella subcapitata* (Chlorophyta*).* This growth inhibition has been attributed to the synthesis of two phenolics, (+)-catechin and pyrogallic acid, while other phenolics (e.g., gallic, ellagic, protocatechuis or caffeic acid) did not affect growth [104].

In another study, the addition of the two flavones apigenin and luteolin to the medium of the cyanobacterium *Mycrocystis aeruginosa* lowered the growth rate in concentration-dependent manner [105]. Also, the addition of the flavanone naringenin to different cyanobacteria cultures induced growth inhibition in six species and no significant effect in two cultures [106]. In all the species naringenin was internalized into the cyanobacteria cell wall structures, while to date the compound naringenin has not been detected in Cyanobacteria [106].

## 4 Conclusions

Enzymatic machinery determining key intermediates in the phenolic compounds synthetic pathway described in algae is almost conserved in all major algal divisions, suggesting a common cyanobacterial origin and an intra-phylum descent, with orthology into mono-phyletic groups. One exception concerns the initiating PAL enzyme that is not recurrent in all microalgal taxa, potentially suggesting alternative routes to initiate the PCs biosynthesis pathway. However, the pathway branches off to multiple directions thanks to a series of multifunctional enzymes as oxidoreductases, that can repeatedly and greatly modify compounds with a common backbone, although different algal groups may differ for the endpoint products.

Endosymbiotic events and species radiation may have later diversified the PCs pattern, diverging toward more complex chemical structures and more specifical functions, e.g., diatom flavonoids, halogenated PCs in seaweeds, algal anthocyanins or algal lignins. Since the little information available on phenolic compounds determination in microalgae, it is not yet possible to draw some hypotheses or conclusion on potential relationship between specific compounds and taxonomical division. This study contributes to improve the knowledge of PCs in microalgae and lays the foundation for future synthetic biology or bioengineering approach to make microalgae a cell factory for the synthesis of bioactive PCs.

## CRediT authorship contribution statement

**Del Mondo Angelo:** Conceptualization, Methodology, Formal analysis, Visualization, Writing – original draft, Writing – review & editing. **Sansone Clementina:** Conceptualization, Writing – review & editing, Project administration. **Brunet Christophe:** Conceptualization, Writing – review & editing, Project administration.

## Declaration of Competing Interest

The authors declare that they have no known competing financial interests or personal relationships that could have appeared to influence the work reported in this paper.
