# Supplementary figures and images for: Insights into the biosynthesis pathway of phenolic compounds in microalgae
Source: Comput Struct Biotechnol J. 2022 Apr 20;20:1901–13. doi: 10.1016/j.csbj.2022.04.019 (PMC9052079; doi:10.1016/j.csbj.2022.04.019)

Tree scale: 1

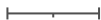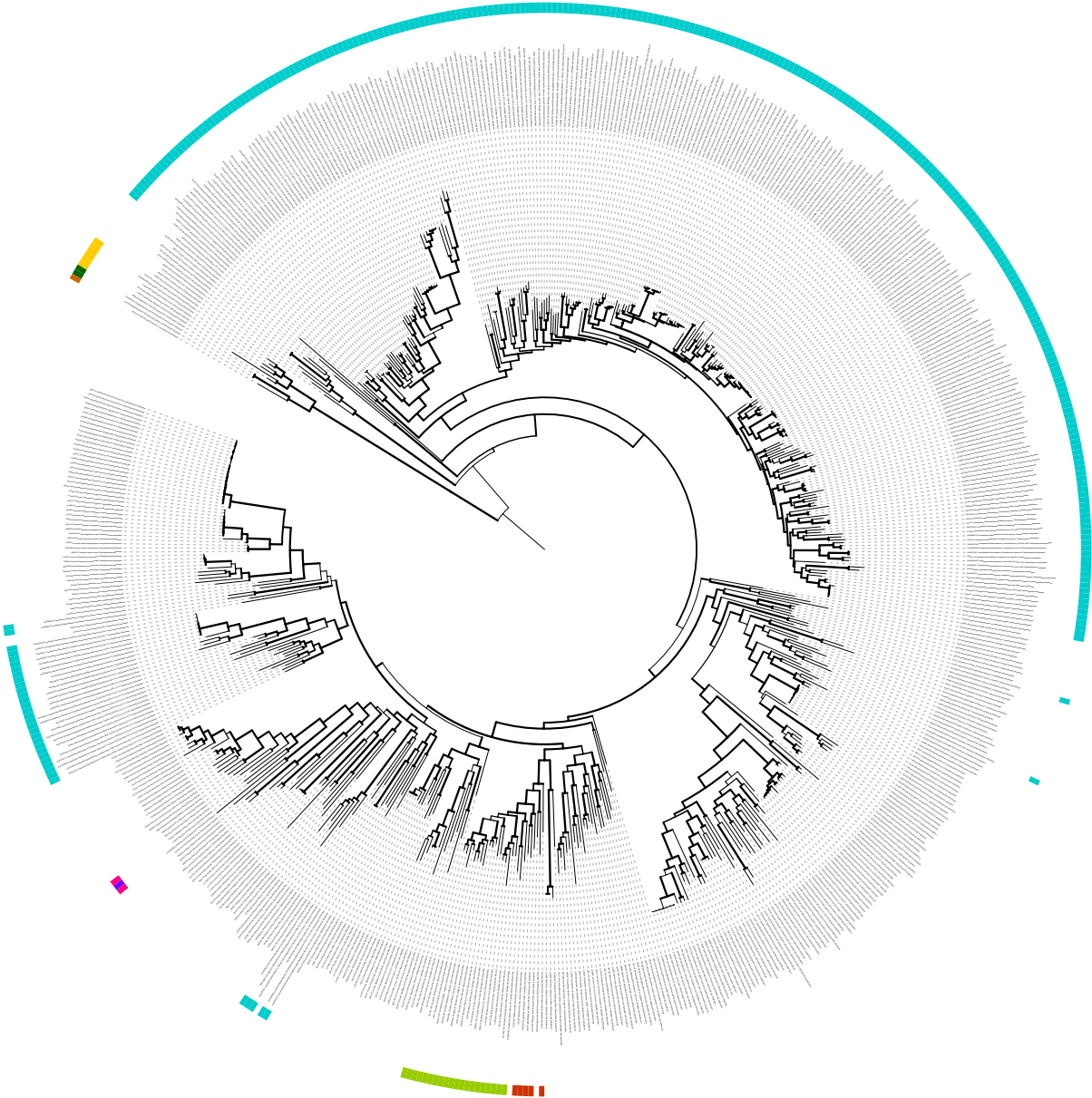

Supplement: Supplementary data 1 [file mmc1.pdf]

Tree scale: 1

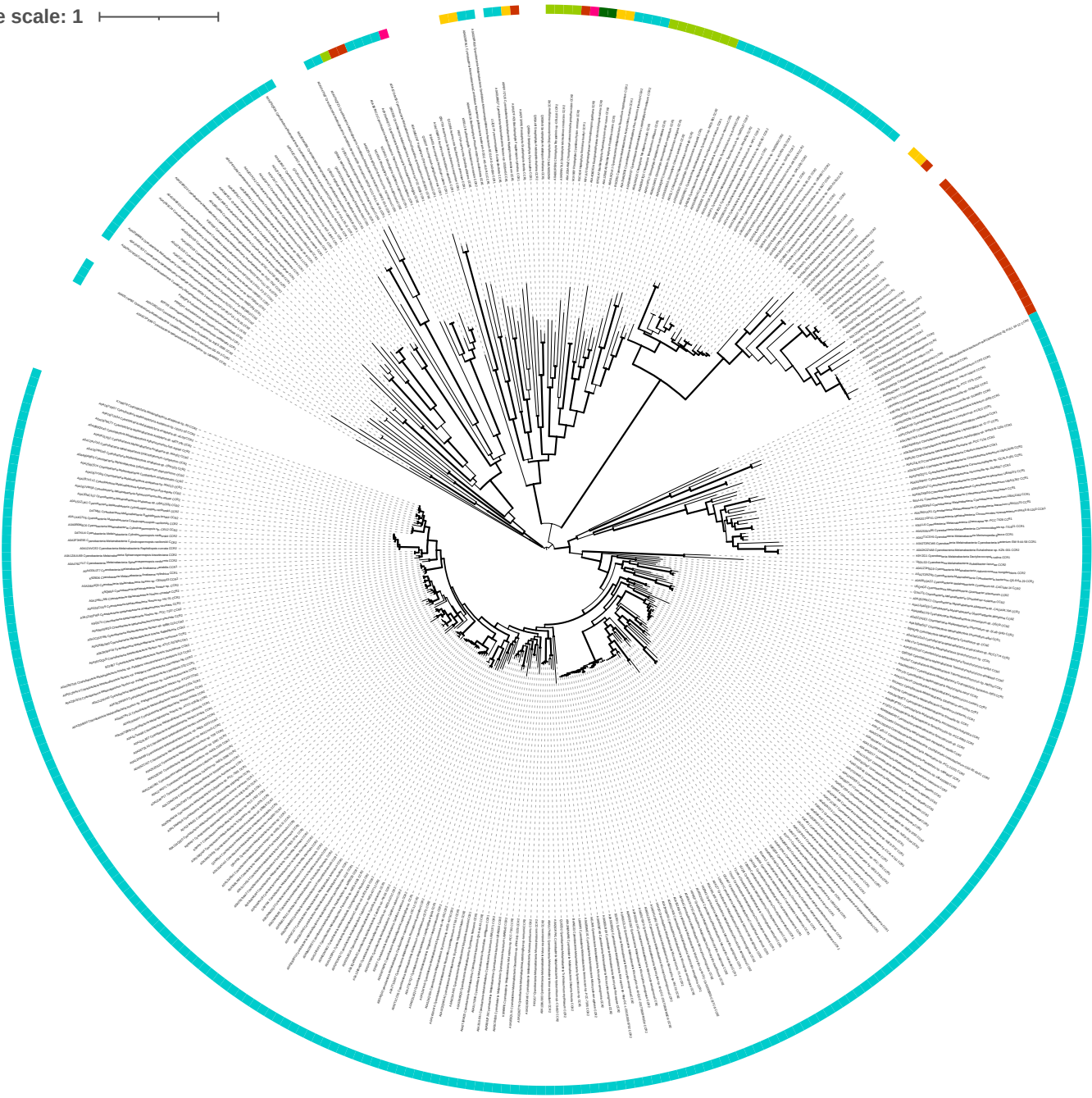

Supplement: Supplementary data 2 [file mmc2.pdf]

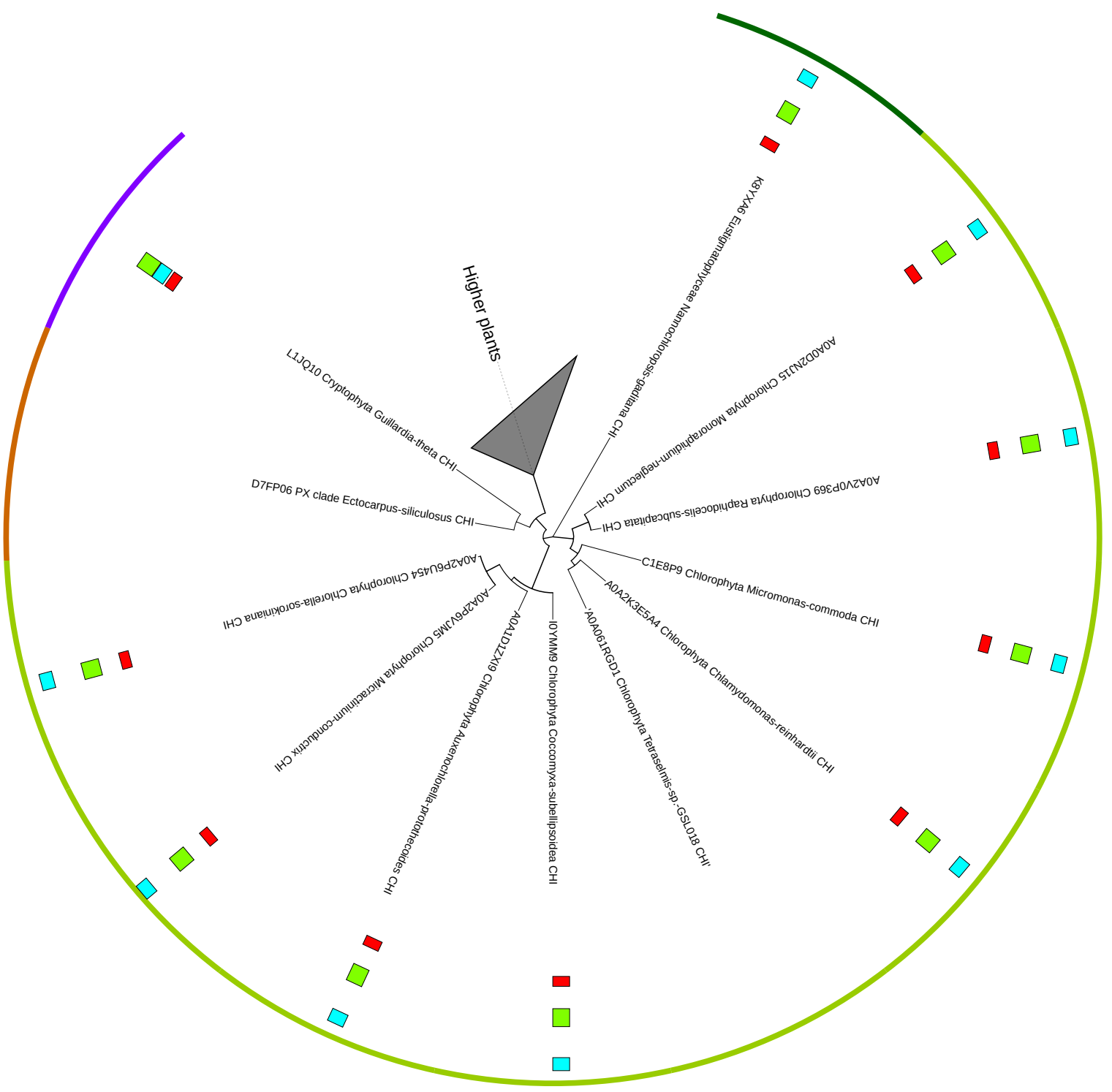

Supplement: Supplementary data 3 [file mmc3.pdf]

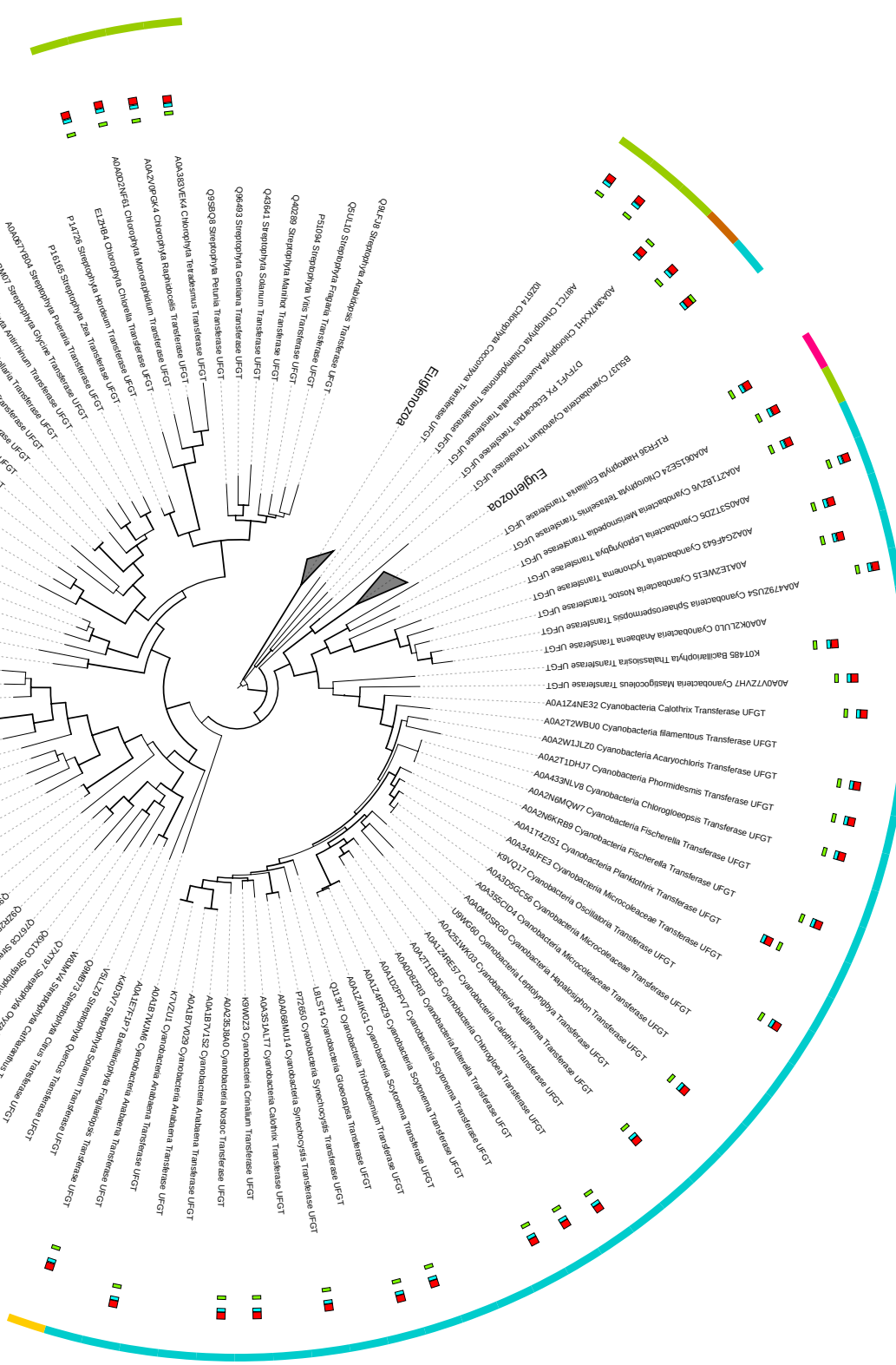

Tree scale: 1

Supplement: Supplementary data 4 [file mmc4.pdf]

Tree scale: 1

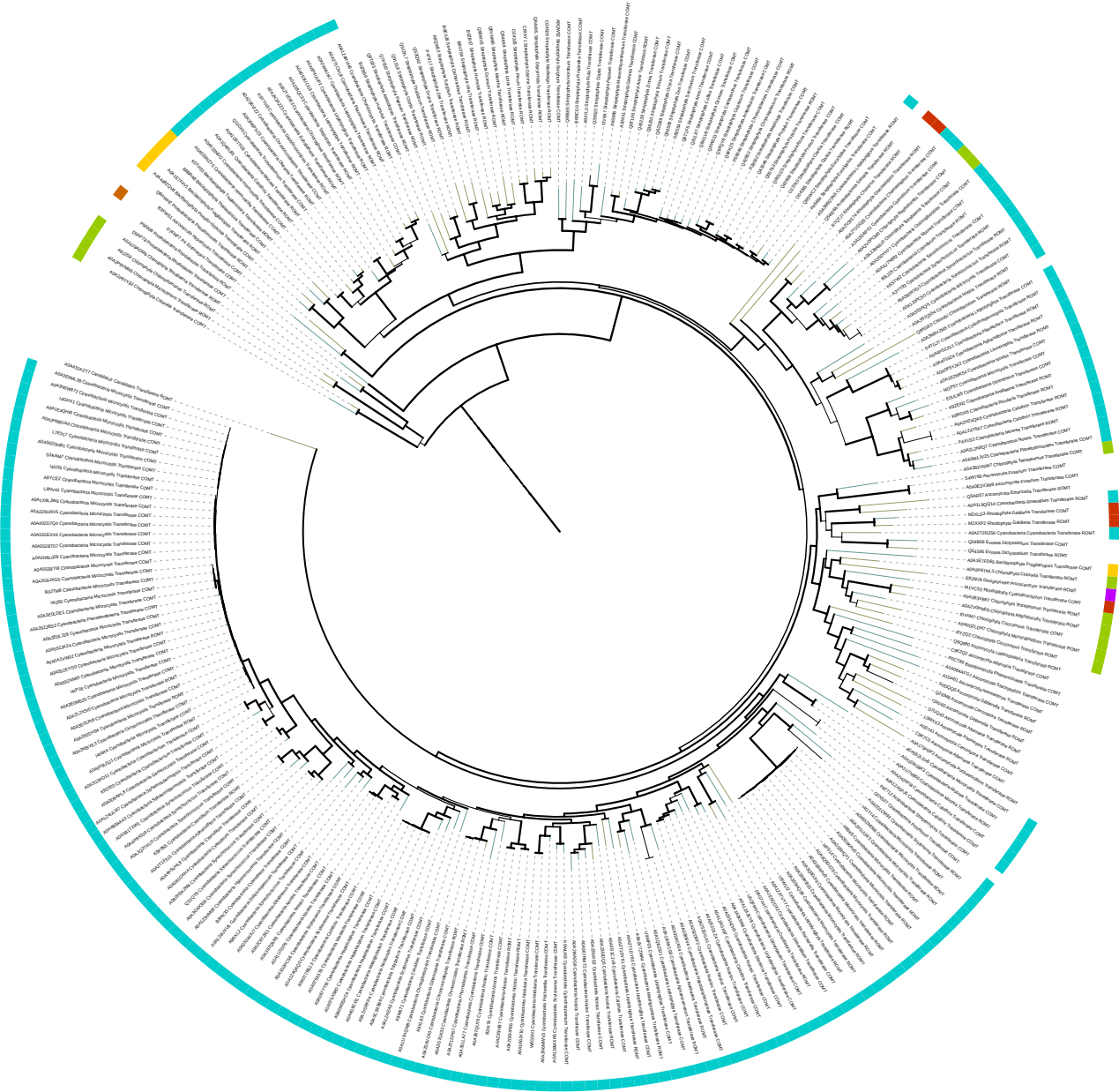

Supplement: Supplementary data 5 [file mmc5.pdf]
