# Supplementary material for: Insights into the biosynthesis pathway of phenolic compounds in microalgae
Source: Comput Struct Biotechnol J. 2022 Apr 20;20:1901–13. doi: 10.1016/j.csbj.2022.04.019 (PMC9052079; doi:10.1016/j.csbj.2022.04.019)
Supplement: Supplementary data 6 [file mmc6.pdf]

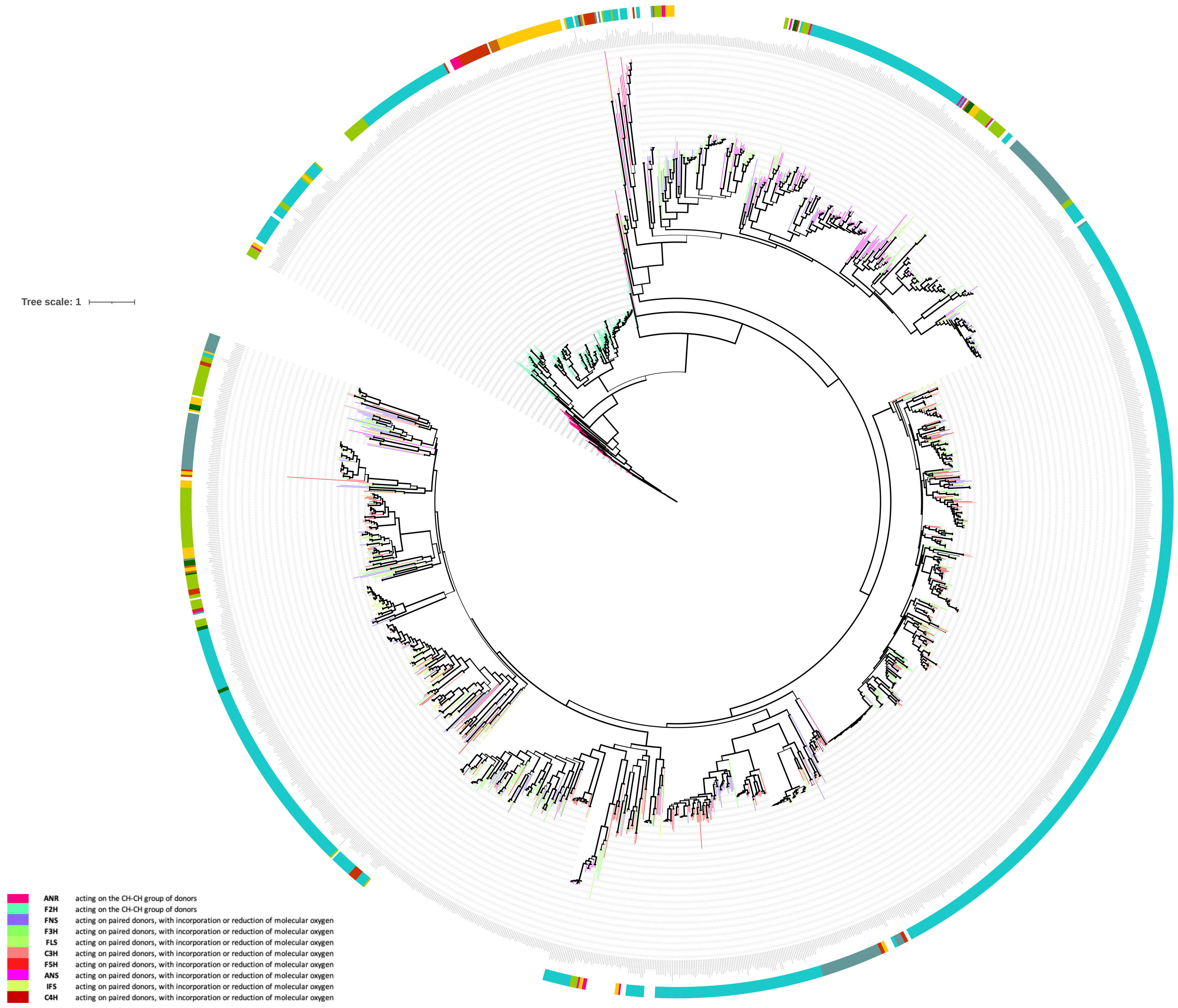

Tree scale: 1
ANR acting on the CH-CH group of donors
F2H acting on the CH-CH group of donors
FNS acting on paired donors, with incorporation or reduction of molecular oxygen
F3H acting on paired donors, with incorporation or reduction of molecular oxygen
FLS acting on paired donors, with incorporation or reduction of molecular oxygen
C3H acting on paired donors, with incorporation or reduction of molecular oxygen
F5H acting on paired donors, with incorporation or reduction of molecular oxygen
ANS acting on paired donors, with incorporation or reduction of molecular oxygen
IFS acting on paired donors, with incorporation or reduction of molecular oxygen
C4H acting on paired donors, with incorporation or reduction of molecular oxygen
