# Supplementary material for: Insights into the biosynthesis pathway of phenolic compounds in microalgae
Source: Comput Struct Biotechnol J. 2022 Apr 20;20:1901–13. doi: 10.1016/j.csbj.2022.04.019 (PMC9052079; doi:10.1016/j.csbj.2022.04.019)
Supplement: Supplementary data 7 [file mmc7.docx]

| **Enzyme** | **Domain ID** | **Domain description** | **Consensus motif** | **Score** | **Positive algal sequences** | **% of positive sequences *** |
| --- | --- | --- | --- | --- | --- | --- |
| F2H | IPR008030 | NmrA-like domain | VIGATGTLG | 7,8E-026 | 114 | 100 |
| COMT | IPR031725 | Acetylserotonin O-methyltransferase, dimerisation domain | YVAAKLGIAD | 1,3E-018 | 40 | 90 |
| CHS | IPR013601 | FAE1/Type III polyketide synthase-like protein | LSGMGCSAGVIGIDL | 5,4E-014 | 26 | 96 |
| CHS | IPR013601 | FAE1/Type III polyketide synthase-like protein | PTPSLSAMLV | 5,4E-014 | 26 | 96 |
| FNS | IPR017972 | Cytochrome P450, conserved site | FGGGARKCIG | 5,7E-014 | 52 | 94 |
| CHS | IPR011141 | Polyketide synthase, type III | IGFMGCAA | 6,8E-014 | 28 | 96 |
| CHS | IPR011141 | Polyketide synthase, type III | AFGPGLSIE | 3,9E-012 | 28 | 93 |
| DFR | IPR001509 | NAD-dependent epimerase/dehydratase | AVTGATGFVGSHLV | 2,1E-011 | 32 | 97 |
| UFGT | IPR035595 | UDP-glycosyltransferase family, conserved site | VPQQAVLSHPAVRAF | 6,5E-011 | 12 | 100 |
| IFS | IPR017972 | Cytochrome P450, conserved site | FGGGPRRCIG | 6,9E-009 | 56 | 95 |
| UFGT | IPR002213 | UDP-glucuronosyl/UDP-glucosyltransferase | ANDQPGNAARIA | 7,3E-009 | 38 | 92 |
| COMT | IPR029063 | S-adenosyl-L-methionine-dependent methyltransferase | DRCZLVGGDFF | 7,9E-009 | 50 | 84 |
| PAL | IPR001106 | Aromatic amino acid lyase | GNFLGQYV | 1,1E-008 | 27 | 89 |
| CAD | IPR002328 | Alcohol dehydrogenase, zinc-type, conserved site | GHEVVGTVAA | 5,8E-008 | 91 | 55 |
| COMT | IPR016461 | O-methyltransferase COMT-type | LASVGIFAE | 6,0E-008 | 51 | 75 |
| COMT | IPR001077 | O-methyltransferase domain | DRCZLVGGDFFE | 8,4E-008 | 50 | 86 |
| CHI | IPR016087 | Chalcone isomerase | GVGVRAKRI | 2,2E-007 | 12 | 100 |
| ROMT | IPR031725 | Acetylserotonin O-methyltransferase, dimerisation domain | LYRVLRALA | 2,2E-007 | 28 | 61 |
| ANS | IPR005123 | Oxoglutarate/iron-dependent dioxygenase | AGAHSDYGSJTL | 1,3E-006 | 52 | 96 |
| ANS | IPR026992 | Non-haem dioxygenase N-terminal domain | GDLKEAFNLG | 2,9E-006 | 48 | 94 |
| ANS | IPR026992 | Non-haem dioxygenase N-terminal domain | RGYTGIERE | 2,9E-006 | 48 | 94 |
| F5H | IPR017972 | Cytochrome P450, conserved site | FGGGLRECJG | 2,9E-006 | 51 | 100 |
| ROMT | IPR016461 | O-methyltransferase COMT-type | AJYVAAELGIFDLL | 3,4E-006 | 37 | 76 |
| ROMT | IPR029063 | S-adenosyl-L-methionine-dependent methyltransferase | EALPQGGR | 8,5E-006 | 37 | 70 |
| F3H | IPR005123 | Oxoglutarate/iron-dependent dioxygenase | ARLSFPFF | 1,1E-005 | 22 | 91 |
| ROMT | IPR001077 | O-methyltransferase domain | LKGVLFDLPD | 1,6E-005 | 36 | 67 |
| FLS | IPR005123 | Oxoglutarate/iron-dependent dioxygenase | RRRYSIPFF | 2,1E-005 | 39 | 79 |
| FLS | IPR005123 | Oxoglutarate/iron-dependent dioxygenase | RSTPHRVL | 2,1E-005 | 39 | 79 |
| CHS | IPR004655 | 3-oxoacyl-[acyl-carrier-protein] synthase 3 | GAGAVILQSC | 2,5E-004 | 131 | 99 |
| CHS | IPR004655 | 3-oxoacyl-[acyl-carrier-protein] synthase 3 | IQAKIGATNA | 2,5E-004 | 131 | 99 |
| CHS | IPR004655 | 3-oxoacyl-[acyl-carrier-protein] synthase 3 | LSILKCLNSLNJS | 2,5E-004 | 131 | 96 |
| CHS | IPR004655 | 3-oxoacyl-[acyl-carrier-protein] synthase 3 | ADKLSIPSNKIISNL | 2,5E-004 | 131 | 99 |
| CHS | IPR004655 | 3-oxoacyl-[acyl-carrier-protein] synthase 3 | DLIVMSGFGAGLTWG | 2,5E-004 | 131 | 99 |
| CHS | IPR004655 | 3-oxoacyl-[acyl-carrier-protein] synthase 3 | LATSTPNDLFGSASQ | 2,5E-004 | 131 | 99 |
| CHS | IPR013747 | 3-Oxoacyl-[acyl-carrier-protein (ACP)] synthase III, C-terminal | SGFGAGFT | 2,5E-004 | 135 | 99 |
| 4CL | IPR000873 | AMP-dependent synthetase/ligase | TSGTTGKPK | 8,3E-004 | 109 | 92 |
| 3-dehydroquinate dehydratase | IPR022893 | Shikimate dehydrogenase family | TKLLGVIG | 2,1E-003 | 107 | 95 |
| C4H | IPR002401 | Cytochrome P450, E-class, group I | ELKDELATJ | 1,7E-002 | 54 | 30 |
| FNS | IPR002401 | Cytochrome P450, E-class, group I | FALMEMKIILATJL | 2,0E-002 | 53 | 26 |
| C3H | IPR002401 | Cytochrome P450, E-class, group I | FAGHETTA | 4,0E-002 | 46 | 26 |
| LAR | IPR008030 | NmrA-like domain | LVIGATGTLGRQVV | 4,2E-002 | 7 | 100 |
| ANR | IPR001509 | NAD-dependent epimerase/dehydratase | LVTGATGF | 5,8E-002 | 28 | 100 |
| IFS | IPR002401 | Cytochrome P450, E-class, group I | GDALAMLE | 7,7E-002 | 56 | 30 |
| CCR1 | IPR001509 | NAD-dependent epimerase/dehydratase | FLPERFGA | 1,8E-001 | 53 | 89 |
| CCR2 | IPR001509 | NAD-dependent epimerase/dehydratase | FLPERFGA | 1,8E-001 | 53 | 89 |
| F5H | IPR002401 | Cytochrome P450, E-class, group I | IPFGGGLR | 6,0E-001 | 38 | 29 |
| F3H | IPR027443 | Isopenicillin N synthase-like | GDMLERMTGGLYKST | 6,2E-001 | 22 | 50 |
